# Practical Cross-Layer Radio Frequency-Based Authentication Scheme for Internet of Things

**DOI:** 10.3390/s21124034

**Published:** 2021-06-11

**Authors:** Arie Haenel, Yoram Haddad, Maryline Laurent, Zonghua Zhang

**Affiliations:** 1 Samovar, Telecom SudParis, Institut Polytechnique de Paris, 91120 Palaiseau, France; maryline.laurent@telecom-sudparis.eu; 2 Department of Computer Science, Jerusalem College of Technology, Jerusalem 91160, Israel; haddad@g.jct.ac.il; 3 Institut Mines-Telecom Lille Douai, 59500 Douai, France; zonghua.zhang@imt-lille-douai.fr

**Keywords:** communication protocol, network security, authentication, internet of things, physical layer, wireless sensor network, sensors security

## Abstract

The Internet of Things world is in need of practical solutions for its security. Existing security mechanisms for IoT are mostly not implemented due to complexity, budget, and energy-saving issues. This is especially true for IoT devices that are battery powered, and they should be cost effective to be deployed extensively in the field. In this work, we propose a new cross-layer approach combining existing authentication protocols and existing Physical Layer Radio Frequency Fingerprinting technologies to provide hybrid authentication mechanisms that are practically proved efficient in the field. Even though several Radio Frequency Fingerprinting methods have been proposed so far, as a support for multi-factor authentication or even on their own, practical solutions are still a challenge. The accuracy results achieved with even the best systems using expensive equipment are still not sufficient on real-life systems. Our approach proposes a hybrid protocol that can save energy and computation time on the IoT devices side, proportionally to the accuracy of the Radio Frequency Fingerprinting used, which has a measurable benefit while keeping an acceptable security level. We implemented a full system operating in real time and achieved an accuracy of 99.8% for the additional cost of energy, leading to a decrease of only ~20% in battery life.

## 1. Introduction

The incredible potential and impact of the Internet of Things (IoT) is a recognized fact. Its rapid growth in recent years makes the task of securing it only more challenging. John Chambers, Cisco Executive Chairman and former CEO, asked “What does Internet of Everything mean for security?” at the beginning of 2015 [1]. Five years later, more and more industries have joined the movement of the global connectivity. Furthermore, it still seems the security solutions lag behind. As presented in [2,3], consumer devices still lack security design and appropriate solutions.

IoT devices are abused in all sort of ways. They may be used as entry points in systems, like the casino that was reported in 2017 as breached through its lobby-connected fish tank and saw its database hacked and 10GB of data stolen [4]. Or they are maliciously controlled for large-scale attacks, like in the case of the Mirai malware [5]: it created a huge botnet that was used to take down several web sites by Distributed Denial of Service (DDoS) attacks. It was able to accomplish that by taking control of hundreds of thousands of IoT devices, especially IP Cameras. More frightening are probably the cyber-physical systems attacks (successful or not) on critical infrastructure lifeline sectors, e.g., water [6], healthcare [7], energy [8].

Several factors make IoT devices the preferred target for attackers, to cite a few [9,10,11,12]. Many of them are cheap devices made by manufacturers who recently added connectivity to their products and have no experience in security; IoT equipment may be designed to stay in the field without a way to provide software or firmware updates, even if some vulnerability has been disclosed, and still, they are connected to the network; some are low-powered, low-resources, low-cost devices and do not implement modern Information Security (InfoSec) methodologies and techniques; hundreds of different platforms and manufacturers lead to a very fragmented market and complicate the design and development of security solutions; a lack of standardization hinders the ability to secure connected devices from different manufacturers.

In the last few years, agriculture technology (agritech) has seen some of the highest IoT adoption levels in comparison with energy, mining and transport [13]. The Office of Homeland Security published an assessment that precision agriculture technology is increasing cyber targeting against the Food and Agriculture (FA) sector, and advised the farming industry to increase awareness, protect their data, and follow some mitigation measures [14]. In domains like agritech, IoT nodes are often low-power, low-cost sensors and actuators. The efficiency factor is of high importance, due to the fact that they may be deployed for very long periods, with technologies like LPWAN providing long-range communications up to 40 km (with future expectations up to 1000 km) and more than 10-year battery life [15]. However, low cost and low power are two limiting factors to the ability of creating secure systems.

In this paper, we propose a hybrid authentication scheme whose main purpose is to save energy at the sensor side.

The organization of this paper is as follows: Section 2 presents the challenges seen today for IoT devices’ lightweight authentication in the context of radio communications. Section 3 introduces a wireless sensors network system and defines the scope of our research. We also define the threat model used for this system. In Section 4, we present our hybrid scheme, with the details of the communication protocol. Section 5 presents an informal evaluation addressing each security property of the threat model. In Section 6, we present an implementation of the system, with evaluation of its accuracy and a precise energy performance assessment. Finally, Section 7 concludes the paper.

Preliminary parts of this work have appeared previously in [16,17], where the protocol scheme was introduced and the methodology of the energy performance demonstrated.

## 2. Related Works and Challenges

In the context described in Section 1, one of the looming concerns is the definition and adoption of lightweight IoT security mechanisms. One of the difficulties is that IoT devices are often designed to accomplish very specific and limited tasks, but as part of complex ecosystems. Their security depends on their ability to defend targeted attacks in challenging and always-changing environments, but with their limited resources. The lack of resources is, according to Curran [18], the reason that “the adoption of security support ecosystems, such as large databases of malware signatures, is impractical”.

Full-fledged security protocols are not always required to achieve a level of “good enough security”, and as stated by Sandhu, “Good enough always beats perfect” [19]. Not all IoT systems require the same strength of protection mechanisms and the same procedures to be deemed secure enough. However, by just proposing *“complete”* security solutions, these become too heavy and are not adopted. For example, in the case of sensors delivering non-confidential information, encryption may not always be required. The additional complexity and price rise due to the additional requirements to add strong encryption support is an obstacle to the adoption by the devices manufacturers. As a general approach to the security of a system, it is important to create an adapted threat model, in order to define the security goals. The security mechanisms put in place will be considered appropriate if they are expected to be effective in addressing those goals.

In this regard, different approaches for lightweight message authentication in resource starving environment have been proposed. Symmetric cryptography solutions include Message Authentication Code (MAC) based on shared secret using block cipher constructions (like CMAC) and hash-based MAC using secure hash function with adequate construction (like HMAC-SHA-256), e.g., [20,21]. However, even if these methods are usually more lightweight than asymmetric cryptography solutions, they are still computationally demanding. In their survey of symmetric lightweight algorithms [22], Biryukov and Perrin call specialized algorithms providing one function with high performance *ultra-lightweight*. This is the case of the lightweight message authentication codes SipHash [23] and Chaskey [24].

Radio frequency fingerprinting (RFF) is the identification of the wireless transmitter based on the analysis of the signal received by a receiver. This identification is based on hardware differences between the transmitters (e.g., tiny imperfections due to the manufacturing process) [25] and on channel characteristics of the transmissions [26,27].

Device RF Fingerprinting has been proposed for a long time to solve the problem of node forgery and impersonation, which “constitutes one major security threat facing wireless networks” [28]. As part of their survey on Authentication Protocols for IoT, Ferrag et al. have reviewed 23 protocols fully or partially addressing the impersonation (*spoofing*) attack [29]; among all these protocols, only [30] presented “a plan of cross-layer authentication using the RF fingerprint of hardware to identify whether messages are from the same wireless device”.

Cryptography-based security requires resources; in most cases, this is computing power. One of the main advantages of a practical protocol using Radio Frequency Fingerprint (RFF) for IoT device authentication is that it would not require computation on the lightweight (and sometimes battery-powered) transmitting device/sensor since the identification computation is mainly done on the receiver side, which is usually less power constrained.

However, such an efficient system is not easy to achieve. In the domain of device authentication, radio transmitter fingerprinting systems were proposed for more than two decades (e.g., [31,32,33]). The authors of [34] provided results of RFF comparisons based on multiple features and of several Machine Learning classification algorithms and reported that they achieved “an overall accuracy higher than 80%, which can be suitable to support multi-authentication of IoT devices”. However, this is not enough to be a viable first (let alone *single*) factor of authentication. Some more recent researches look very promising, even if still in “conceptual development” stage, and the ones reporting to achieve 99% accuracy were so far only under ideal conditions [35]. In more realistic environments, the accuracy is degrading quickly. For instance, lower Signal-to-Noise Ratio (SNR) and factors like Line-of-Sight (LOS) vs. Non-Line-of-Sight (NLOS) directly affect the precision of the identification systems [36,37].

Therefore our challenge is to make use of the existing schemes, despite their relative low accuracy, and still benefit from the fact that they do not need computation from the transmitter, while at the same time, it does not compromise the system security.

## 3. Network System and Threat Model

In order to adhere with the “good-enough-security” principle as described in Section 2, we must define the set of required security properties that will respond to the security threat model of our system. We use a concrete context, and as an example of embodiment, we define our system as similar with current implementations of smart (or precision) agriculture systems. Examples of properties measured in this context are numerous, including temperature, humidity, acoustic, proximity, acidity, motion, etc. These measurements are used to evaluate weather conditions, soil quality, pathogens, insect pest detection, crop’s growth, drug residues, heavy metal, etc.

### 3.1. Wireless Sensor Network System Definition

**Figure 1:**
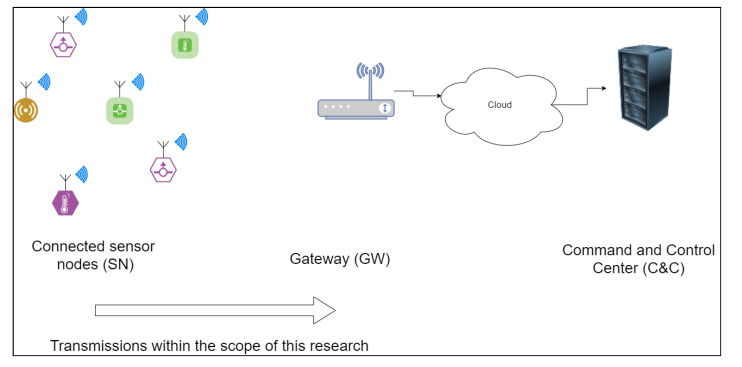
System model description.

We define the system as a wireless sensor network, consisting of wireless, spatially distributed, fixed-location sensor nodes and a gateway. The purpose of the sensors is to transmit real-time measurements to a command and control (C&C) center, via the gateway (Figure 1). The sensors are battery powered and should be as cheap as possible in order to be deployable in numbers. As such, they should implement only the minimal subset of required functionalities.

We present our method using the case of a wireless sensor network (WSN) comprising unattended sensor nodes (SN) transmitting short messages to a gateway (GW). Each SN consists of at least a battery powered micro-controller, a transceiver and some sensors. Each sensor is initialized with an identifier (ID) and a pre-shared key (PSK), which is a secret value used for cryptographic authentication before being deployed. Both the ID and the PSK are known by the GW.

We assume that for such a system, only short messages are of interest, and therefore, the protocol is optimized for this matter.

### 3.2. Threat Model

There are a few security assumptions for this system:

- Key provisioning. We assume that a pre-shared key (PSK) has been serialized in each node and is known only by the gateway. The method for this provisioning and physical attacks on the nodes are out of the scope of this research.
- Physical attacks. Depending on the system, the nodes can be hardened or not accessible. Physical attacks, e.g., extracting the PSK from the node, are not in scope of this research and not part of its threat model.
- Practically unfeasibility of purposely flipping certain bits by jamming. Jamming is usually aimed at radio signals to disrupt the reception of the original transmission by a receiver. In theory, it would be possible to purposely flip some bits of the transaction, but we consider such an attack *practically* unfeasible [38]. This also means that we can assume the equivalence of the authentication of a RF message source to the authentication of its content.
- Robustness of the hash function used. For our analysis of the scheme, we will consider the cryptographic hash function to be computationally secure against first pre-images, second pre-images and collisions.

The threats taken into account for this system, and thoroughly analysed in Section 5, are:

- Message forgery
- Message replay attack
- Message origin impersonation
- Man-in-the-middle attack

## 4. Hybrid Cross-Layer Authentication Protocol Scheme

### 4.1. Overview of the Scheme

In this work, we propose a hybrid cross-layer authentication protocol that tackles the aforementioned challenges, namely reducing the energy consumption of low-resource devices, by leveraging known RFF technologies together with known lightweight cryptographic authentication algorithms. The objective is to achieve this goal even if the actual RFF techniques as used today are not yet on par with the security level of other authentication techniques. The authentication of a single message may be achieved through the RFF or through cryptographic authentication. By using both approaches in the same protocol, not as multi-factors of the authentication system but as complementary methods, we create a Hybrid Authentication mechanism: each message sent by SN to GW is authenticated *or* by RFF *or* by cryptographic authentication (in the case the RFF message authentication failed).

Our main goal is to transmit the messages in an authenticated way, with the minimal impact on the power consumption of the SN. We define and propose using a hybrid cross-layer authentication protocol. This model will use a standard cryptography based authentication mechanism, along with an RFF identification system, to achieve a lightweight authenticated communication.

### 4.2. Radio Frequency Fingerprinting Calibration

Like biometric-based authentication systems, RFF-based authentication systems need some metrics in order to be compared between them and calibrated. The probabilities of incorrect outcomes of an authentication session are known as False Reject Rate (FRR), sometimes called Insult Rate, and False Accept Rate (FAR), also called Fraud Rate. False Reject means that a rightful client was rejected during the authentication process (e.g., Alice was not authenticated as Alice). False Accept means that someone other than the rightful client was authenticated (e.g., Bob impersonated Alice). Biometric systems (or RFF systems) can be calibrated to be more lenient on the authentication, thereby lowering the FRR. However, the negative side effect is then that the FAR will also increase. On the contrary, raising the threshold so that the FAR is reduced yields a higher FRR. The Equal Error Rate (EER) is obtained by calibrating the threshold so that FAR is equal to FRR. EER is usually used to compare the effectiveness of different systems. Still, this approach is adequate for comparison of mechanisms used for authentication. Rather than using a calibration based on EER, the RFF part of our method is calibrated using an accepted and sufficiently low FAR, based on the level of authenticity requested. As we have seen, this will yield a much higher FRR. In order to deal securely with these false rejects, the receiving side (the Authenticator) will “fall back” on to our secondary authentication system.

In the following embodiment of the scheme, we use a lightweight Challenge—Response Algorithm, based on a keyed-hash using the secret stored in the SN as key. This will be detailed in the next sections.

### 4.3. Challenge–Response Authentication and Message Authentication

**Figure 2:**
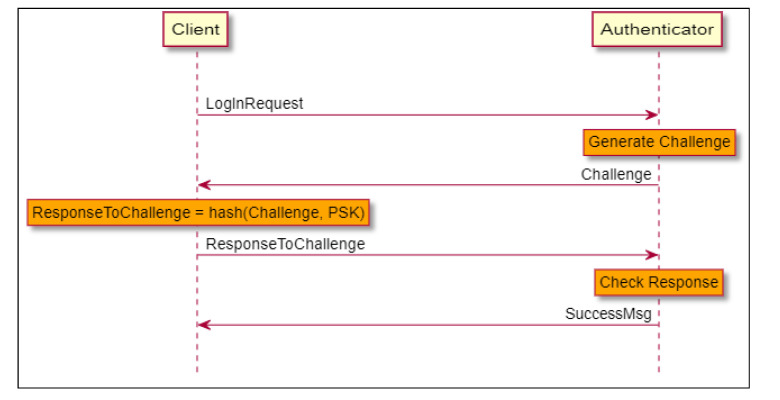
Legacy Challenge–Response authentication session.

We first describe how a system answering our requirements and using some legacy handshake protocols would be built. In Figure 2, a legacy three-way handshake Challenge Response Authentication Protocol session is shown. As described in Section 3.1, both sides share a pre-shared key (PSK). When the client requests to be authenticated, the server (authenticator) sends it a challenge. The challenge itself is a cryptographic nonce, i.e., an arbitrary number used only once. It is generated using a deterministic random bit generator (DRBG) seeded with a monotonic counter to avoid a repetition of sequence.

Both sides calculate the response, which is the cryptographic hash of the challenge concatenated with the PSK. The client sends its response, and the server compares it to its calculation. If they match, the authentication is successful. Several well-known authentication protocols are based on this model, e.g., CHAP [39], EAP [40], and HOTP [41].

As this type of protocol is based on some cryptographic hash, for short messages we can also add information to be sent (e.g., the measurements taken by the sensor), as part of the transaction. Since each transaction and each computation requires time and energy, binding the message with the handshake will minimize the cost of Node Authentication and Message Authentication, in the case the Node (re)-Authentication is needed.

In order to bind the session with the client, a “session token” or “cookie” can be used to avoid some attacks based on stealing the session. This is common practice to avoid session-hijacking attacks [42]. In order to be used as a stateless protocol, i.e., no session information is kept by the server, the system may use a token/cookie that binds some secret known by the client (like some cryptographic key) with the cookie, that may be encrypted by the server key.

In the following sections, we refer to such a protocol based on a cryptographic challenge-response handshake binded with the message and its authentication code as *MAC-only* solution (in contrast with our hybrid solution).

### 4.4. The Benefit of RFF Combined with a Challenge–Response Authentication

A legacy solution such as the one presented in the previous section leads to computation on both ends. In the context of lightweight IoT, we especially try to reduce the computation from the client side. By binding the session token with the perceived RF features from the server side, the client side does not need any new computation to prove its identity, since the binding is transparent and part of the communication itself, like the human voice of a known person.

In the case of sensors sending non-confidential messages, all the security properties may not be needed, and we want to check authenticity of sender but may want to reduce the power consumption and complexity induced by cryptographic computation, handling of secret keys and sessions. As seen in Section 2, this may be considered “good enough security” and more likely to be integrated in practical solutions.

Our approach has the advantage that the RF fingerprint can be used to authenticate the message to the identity of the sender without the need for cryptographic primitives. It makes the communication of short messages resistant against impersonation attacks (“spoofing attacks”) with a minimum effort by the sender.

It has also the advantage of being forward-compatible with future advancements in the field of RFF: since the protocol is agnostic to the RFF method used, it is possible to replace the RFF part of the system by a more evolved one, without having to replace or even update the deployed sensors, since the RFF is integrally implemented on the receiver side. Furthermore, even if the system as a whole is made more power efficient by using a better RFF system, it can make a *practical* use of actual RFF systems, even with their relatively high EER.

### 4.5. Hybrid Authenticated Lightweight Communication

For a Hybrid Authenticated Lightweight communication based on RFF, we consider the case where a single transaction consists of the authenticated delivery of a message including at least some basic functional parts: the SN ID and the payload (for example, sensor values, like temperature, humidity, etc.).

We have to distinguish between the case where RFF was accepted as matching the stored RFF relevant to the SN ID and the case where it is not. As explained before, a system where it is possible to authenticate the messages without the need for some computation and additional information from the SN is preferred in order to reduce the SN energy consumption.

#### 4.5.1. Successful Authentication by RFF

**Figure 3:**
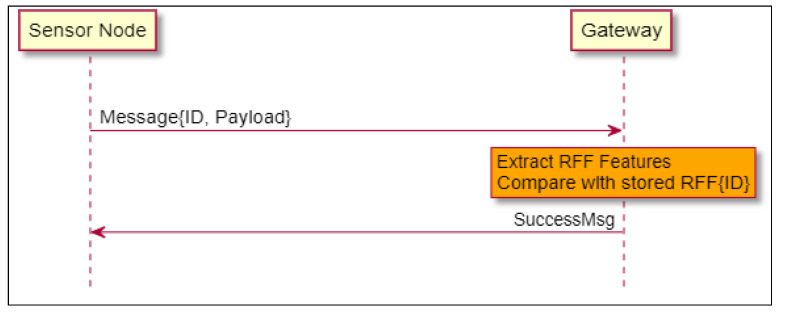
Successful authentication by RFF.

In Figure 3, the message was successfully authenticated by its RFF alone. No other message is needed from the SN.

#### 4.5.2. Unsuccessful Authentication by RFF and Fallback to Cryptographic Primitive

**Figure 4:**
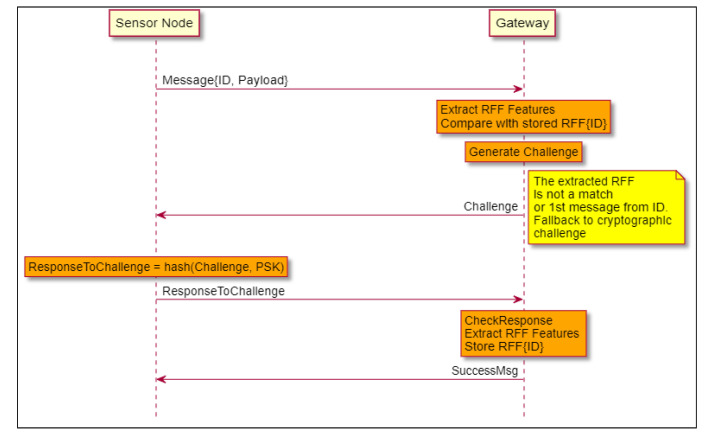
Unsuccessful Authentication by RFF and fallback to CHAP.

In Figure 4, the message was not successfully authenticated by its RFF. This can happen in several cases: the SN has never been authenticated before, and so, the GW never stored its RFF; the RFF was not matched; and of course, it may happen in the case of an adversarial (malicious) transmission.

In order to differentiate between these cases, the GW will issue a challenge to the SN (usually, just a random number), as explained in Section 4.3.

The SN has to answer the challenge by sending the keyed-hash based message authentication code (MAC) of the challenge. The secret used as the key of the MAC is the shared secret as defined at the beginning of Section 4. In order to securely couple the message to the authentication part, the *ResponseToChallenge* message also includes the ID, the payload and the MAC calculated on all the fields to authenticate: challenge, ID and payload, with the Secret as the key.

If the *ResponseToChallenge* is successfully verified by the GW, the RFF features are extracted and the RFF is stored for this specific ID (RFF{ID} in the Figure 4). This scenario is a valid one even if not in the case of a malicious attack. It can happen even for static sensors for example if the environment changes. This demonstrates the self-recovery property of the protocol.

## 5. Informal Security Evaluation

In order to evaluate that the security requirements as defined in Section 3.2 have been addressed, we discuss each threat and review how it is mitigated with our system for both MAC and RFF.

### 5.1. Message Forgery

Message forgery would mean that an attack is be able to forge a single message and have it accepted by the gateway as genuine. This risk does not apply in our system for a single message, since, when using MAC, each message is authenticated, and we assumed the equivalence of the authentication of a RF message source to the authentication of its content (see Section 3.2).

### 5.2. Message Replay

Message replay attack would mean that an attacker could resend a previous message sent by a sensor to the gateway. This risk is mitigated by the fact that using MAC, the message is coming along with a challenge–response authentication, and this does not allow a message to be replayed out of order. Furthermore, using the RFF, the gateway would detect that the message is not coming from the same source.

### 5.3. Message Source Impersonation

An attacker sending a single message in place of the GW is of no interest in our context (except for the case covered in the next section). Furthermore, regarding a sensor impersonation, in our system, a direct impersonation is not possible. Based on the PSK saved in the node, it would be computationally impossible for an attacker to fake the challenge–response based on the MAC (which would be equivalent to break the cryptographic hash function). Furthermore, the RFF is used to authenticate the sensor. As discussed, the configuration of the system has to be done so that the FAR is low enough to meet the security requirements of the given system, as is the case for any biometric authentication system. However, we discuss in the next section how a certain setup could lead to a sensor impersonation through an elaborate attack, if not mitigated.

### 5.4. Man-in-the-Middle Attack

A man-in-the-middle (MITM) attack consists of an intruder relaying messages between both parties of a communication. In our case, an intruder capable of intercepting the messages between the SN and the GW during the CHAP cannot alter the messages, since the GW would detect that the response is not the one it calculated. However, if the intruder is capable of sending the messages in place of the SN, and of blocking the SN messages themselves from reaching the GW, then its RFF would be the one recorded by the GW as the valid one in place of the legitimate SN. The way to achieve this in our context would be for an attacker to jam the messages in a way that he can record the message without letting them reach their destination, and resend them as is from its own transceiver. This attack is similar to others found with systems sending one-way authenticated messages based on rolling codes. This was demonstrated in the so-called “RollJam” attack [43].

The “cryptographic way” to mitigate it is to cryptographically authenticate each message. However, this goes against the very essence of our requirements, which are to save as much of the computation as possible on the sensor side. We therefore would consider other system-level mitigations in place: e.g., first, CHAP executed in safe environment to create an RFF baseline and/or use of an intrusion detection system (IDS) to detect signal jamming in the area of the system. Different implementations of jamming detection techniques have been published and analyzed with great success [44,45,46]. This would solve the MITM attack described here but is not in scope of this paper.

### 5.5. Security Advantage of the Hybrid Approach

A significant advantage of our method is the synergy of some of the methods used, in a flexible manner. Enforcing at the gateway side that one in every *n* message will request a full reauthentication challenge and check for methods at the same time increases the security bar significantly by mixing the advantages of both approaches. The periodicity of the forced reauthentication is flexible and is of course a trade-off between cost and security. For example, against anti-replay attack, the cryptographic approach assumes that the node key was not extracted or lost and uses nonce for the challenge, so that even if an attacker recorded all the previous *Challenge* and *ResponseToChallenge* messages, it cannot replay a valid one for a new challenge. This is a type of intrusion detection and prevention system. However, by having the same property of intrusion detection implemented by a different manner (RFF) and having the system check that both approaches are used at least once every *n* messages, we gain detection capability in the case some attacks find a way to bypass one of our protections, which gives more robustness to the system.

## 6. Scheme Experiment and Evaluations

In this section, we present a full implementation of the system, an accuracy evaluation, and an energy performance evaluation.

### 6.1. Evaluation System Description

From an end-to-end perspective, the evaluation system consists of several sensor nodes communicating with a computer. One of the node is a legit SN, which sends “sensitive” messages, while the others are “rogue” nodes, which send similar messages. The role of the computer (“GW”, since it is playing the role of the GW in our protocol) is to differentiate between the legit one and the rogue ones. The legit SN stays at a fixed location in an outdoor environment, as is the case for numerous scenarios, as explained in Section 3. The rogue nodes may be fixed or mobile.

#### 6.1.1. Original Real-Time RFF Authentication System

In an effort to experiment and evaluate end-to-end the whole scheme on a *live* system, we implemented an authentication system based on Received Signal Strength Indicator (RSSI) values from different anchors. The choice was driven by the fact that most, if not all, modern RFF systems, like the ones cited in Section 2, required post-processing.

We present here an original evaluation system fully working in real-time, inspired by previous works from different domains: using RSSI measurements to evaluate the location of a transmitter is a common technique based on radio triangulation, for indoor location tracking or for geolocation in GPS-degraded environments [47]; leveraging RSSI-based location fingerprinting as part of authentication schemes has been proposed in the past for smartphones or in the context of WLAN [48,49]. It has also been proposed as a second factor of authentication, in the form of a proximity check [50]. By building on top of these methods to create a lightweight RFF-based authentication system, we were able to create an end-to-end evaluation system close to a real-life setup.

It should be noted that switching the RFF measurement part of this system to use another RFF method is straightforward: the sensor implementation, the gateway algorithms and interface control messages rest unchanged, and only the radio-based authentication varies between the system. Furthermore, the RF-based authentication part does not affect the implementation of the sensors themselves, since it is only implemented in the GW (and optionally some processing can be carried out by the C&C). This is by itself one of the advantages of our scheme, as explained above.

As a side note, the authors would like to remark that the RSSI-triangulation based RFF mechanism used in this evaluation was developed as a simple one for a close-to-real-world evaluation of the full system end-to-end, including energy consumption comparisons. However, we would clearly recommend for any manufacturer to choose a more evolved RFF system for production, especially since the method we propose allows an easy switch of the RFF system part without needing any change in the sensors or the communication protocol. Nevertheless, even if it was not the authors’ original intention, it turned out that this “simple” system is quite an efficient and rather precise one.

#### 6.1.2. Selected Cryptographic Primitive

As the Message Authentication Code (MAC) of the cryptographic part of our system, instead of the HMAC construction using a generic hash algorithm like SHA256, we adopted the Chaskey keyed-hash algorithm [24], designed to be fast on short messages, as proposed for lightweight message authentication code by [22] (see Section 2). The Chaskey-12 variant was used, as recently standardized by ISO/IEC [51], which has a very small memory footprint. Another valid choice would have been LightMAC, but with a higher memory cost [52].

For our hybrid solution, in the case of an RFF authentication reject (*Challenge* message), we could have switched back to the same method of just key-hashing the message, but since, in this case, another message was to be sent anyway from the GW to the SN, we chose to use a stronger Challenge–Response protocol based on the same MAC primitive, without any additional effort. In this way, we gained protection against Replay Attacks, without the need to keep a monotonic counter at both ends and without any more computational overhead.

### 6.2. Accuracy Evaluation

In this section, we evaluate how our hybrid system enhances an RFF scheme in order to achieve a much better accuracy.

#### 6.2.1. Testbed of the Accuracy Evaluation System

**Figure 5:**
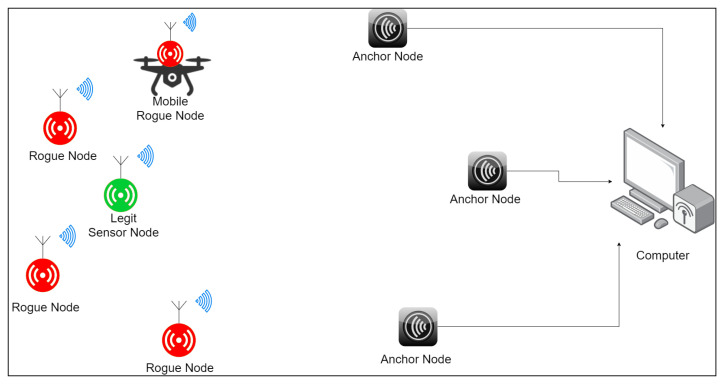
Evaluation system setup.

The receiving side includes three RF anchors placed in different locations. They all receive the messages sent by the nodes, and at the same time measure the RSSI. This value, along with the message, is sent to a computer. Each message sent by the sensors has a unique ID, so that it is possible to synchronize the values received by the anchor nodes. Therefore, for each message received, the computer also receives three RSSI values. These values are used to create a simple RFF of the sender. A machine learning algorithm is then used to identify and validate the sender based on this RFF. In the case the identity has not been validated based on the RFF, the computer sends a *Challenge* message and follows the the protocol described in Section 4.5.2. A high-level schema of the system is presented in Figure 5.

**Figure 6:**
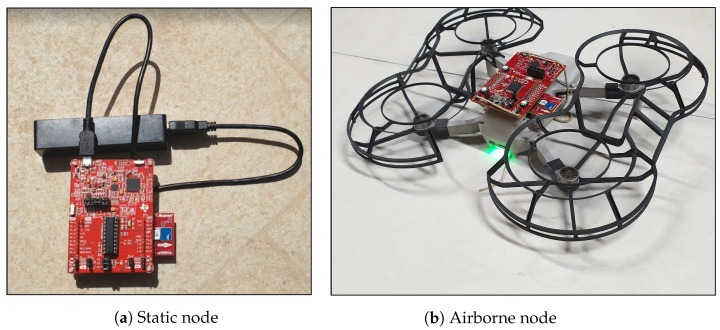
(**a**) MSP-EXP430G2ET, powered by an external battery pack. (**b**) MSP-EXP430FR5994, mounted on a Mavic Mini drone. Both use a CC110L RF BoosterPack.

The nodes are all based on three different types of Texas Instrument ultra-low-power development boards, all powered by TI MSP430 RISC micro-controllers. The RF module is the CC110L transceiver, using the 868–870 MHz industrial, scientific, and medical (ISM) radio band. The CC110L transceiver is integrated using the Anaren CC110L RF BoosterPack, a low-power wireless transceiver extension kit compatible with the MSP-EXP430 (Figure 6a). The anchor points use the same radio modules as the sensor nodes, mounted on TI TM4C1294 Connected LaunchPad Evaluation Boards.

The evaluation was conducted during the lockdown period of the COVID-19 crisis. As such, it was necessary to find a solution to the limitation of movement, but still to be able to take measurements from different positions. For this purpose, we used a drone that was able to carry some nodes and simulate rogue message attacks from multiple locations, even in three-dimensional space. This turned out to be a very efficient way to test the system.

The legit SN and two rogue nodes, including the drone-mounted one, each used an MSP-EXP430FR5994. The choice was due to the fact that it could be easily mounted on the drone, since it can be powered by means of an on-board super capacitor instead of an external battery [53] (Figure 6b). Other rogue nodes used MSP-EXP430G2ET and were powered by an external battery pack (Figure 6a).

**Figure 7:**
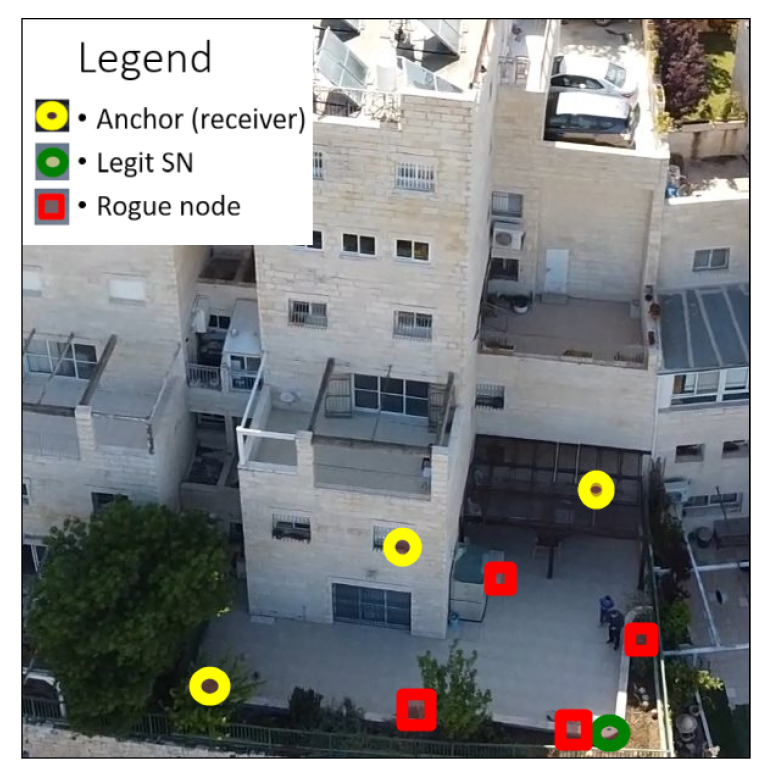
Photography of the live setup of the experiment, taken from the airborne rogue node.

The legit SNs were positioned 12, 9, and 8 m away from the anchor nodes. The airborne rogue node was guided in different positions in the air, from 0.5 m up to 25 m from the legit SN, at different angles from the anchors. The other rogue nodes were placed in different fixed positions, the closest being 30 cm from the legit SN (Figure 7).

#### 6.2.2. Accuracy Evaluation System Results

The RSSI value returned by the CC110L RF module of the anchor nodes is an estimate of the signal power level, based on the current gain setting in the RX chain and the measured signal level in the channel [54].

For each message sent by an SN and received by the anchor nodes, each anchor retransmits, via wired Ethernet connection to the computer the received message along the precise RSSI measured by its radio module. The computer determines the source of origin of the packets based on the RSSI triplets, as explained below.

**Figure 8:**
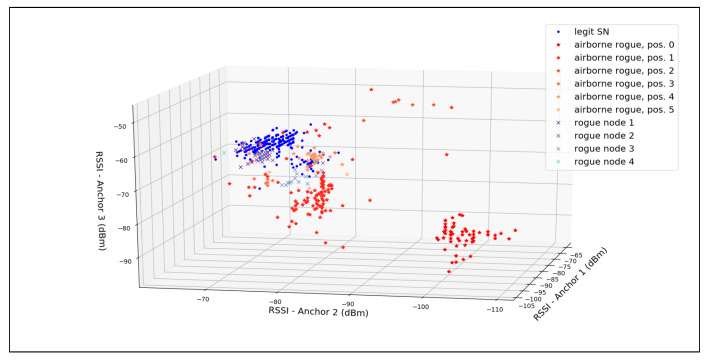
RSSI-triplets measured for 500 valid messages.

In Figure 8, each point represents the triplets of the RSSI values measured by the anchors for a single message. We can see the different clusters of points colored for each transmitting node. Based on these values, we can use a classifier to identify the source of future messages.

The authors of [55] discuss ML for RFF based identification, reaching good accuracy using bagged tree and weighted k-nearest neighbors (KNN) algorithms. Based on the observations made about Figure 8, a natural choice for our evaluation would have been a weighted KNN classifier. Since our goal was to be able to change the configuration in order to reach different values of FAR and FRR, we use a regression KNN model, whose output value will be compared to a threshold value. If, for a certain input, the output of the KNN regressor is greater than the threshold, the message is considered as coming from the valid SN. The threshold value is chosen to have an acceptable FAR, as decided by the system requirements. For an RFF-only system, the accuracy is calculated as ${Accuracy}_{RFF}=1-\left(FAR+FRR\right)$, while for the hybrid system, since a reject falls back to a trusted cryptographic method, the total accuracy will be ${Accuracy}_{Hybrid}=1-FAR$.

**Figure 9:**
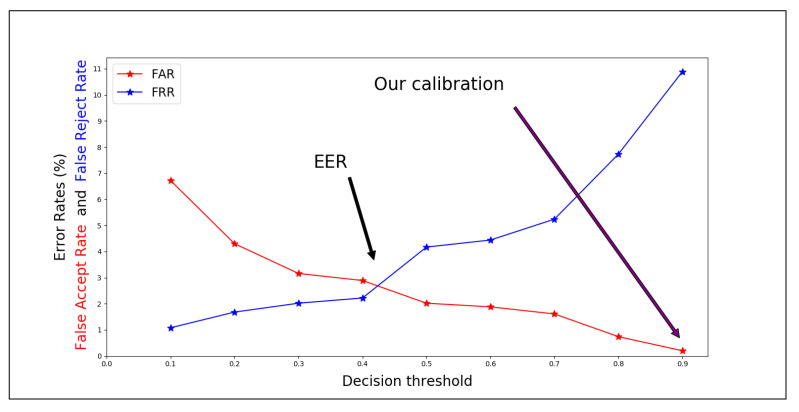
Evaluation RFF calibration by low FAR.

For the evaluation, all the nodes sent messages every second. For the training part of the KNN, the GW answered to all the messages received by a *Challenge* response, thereby assuring the source of the messages even without the RFF. The first 500 valid messages (i.e., those with a valid CRC received by all three anchors) wer used as the training set by the KNN regressor, with K set to 10, and from this point on, the GW followed the hybrid protocol. Figure 9 shows how the threshold influenced the FRR and FAR of the system for 2000 messages. By choosing a threshold > 0.9, we reached a FAR = 0.20% and FRR = 10.89%. Therefore, even though the bare RFF system gave 88.91% of accuracy, the hybrid protocol reached 99.8% accuracy, far better than the other alternatives of pure RFF systems.

### 6.3. Performance Evaluation of Energy Efficiency

In the following, we present a performance evaluation that compares the hybrid scheme as described in Section 6.1 with two approaches: a cryptographic protocol using only a Keyed-Hash Message Authentication Code (*MAC-only*) to authenticate each message, similar to the legacy HMAC [56], and an RFF-only system.

#### 6.3.1. Selected Protocols Description

The MAC-only nodes implementation makes use of the same Chaskey-12 algorithm defined in Section 6.1.2 in order to authenticate each message sent. The rationale behind the choice of using a MAC-based protocol is that the MAC does not require any other message (unlike a full CHAP) and can be seen as one of the most lightweight cryptographic-only authentication approaches.

In the case of a message received from the MAC-only SN, the GW only checks the MAC validity and sends back the *SuccessMsg* message. In the case of a message from the hybrid protocol SN, a *Challenge* response or a *SuccessMsg* was issued as response to the *Message*, according to a simulated FRR of 10%. This value was chosen as it led to a FAR of 0.2% in our live experiment. It should be noted that according to recently published studies, an FRR as low as 10% would lead to a much better accuracy of our system and a FAR of $∼10^{-9}$ according to the results of recent RFF techniques, as demonstrated by [35]. To complete the evaluation and present how future improvements of RFF technologies can lead to better energy saving, we also present the results of the measurements with an FRR of 5% and, as a theoretical limit, the power consumption of an RFF only system.

#### 6.3.2. Testbed of the Energy Efficiency Evaluation System

To evaluate the energy efficiency of our authentication schemes as presented in this work with equipment simulating IoT SN, we used the Texas Instrument ultra-low-power microcontroller MSP430G2x and the sub-1 GHz RF transceiver CC110L, as in Section 6.

In order to achieve precise measurement, EnergyTrace (a Texas Instrument technology) was used, by means of the MSP-EXP430G2ET Launchpad Evaluation Kit and Code Studio Composer (CCS) [57]. In order to precisely calculate the energy profile, EnergyTrace makes use of an on-board DC–DC converter, which generates the power for the target. The pulses of the converter are counted by the software controlling the converter, and the measurements are acquired through CCS.

The testbed consists of two identical kits. The first one runs the cryptographic-only baseline protocol (MAC-only). The second one runs a complete implementation of the hybrid protocol. The SNs transmit a data packet (the temperature) every 3 s to a GW; this is much faster and more energy-consuming than real-life scenarios. This also prevented the nodes from using the “deep-sleep” mode of the modules between two transmissions. However, is it adequate to compare the two models, with less time spent on the simulation. The energy consumption is measured continuously for each SN.

**Figure 10:**
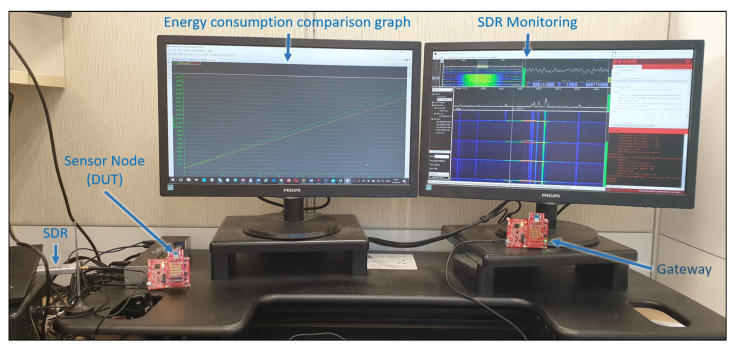
Performance evaluation lab.

The GW runs on a similar platform, but implementing the server side of the protocol. Since our goal is to reduce the energy consumption of the SN, while the client side implements a real implementation of our protocol, the GW simulates the RFF, based on our results from Section 6 and the different values to be compared (simulated FRR of 10%, 5%; and 0% for the simulated RFF-only one). This test bed is shown in Figure 10.

#### 6.3.3. Energy Efficiency Evaluation Results

Table 1 presents the comparison of the energy measurements for the MAC-only (baseline) protocol and the hybrid protocol, calibrated for a FRR = 10%, FRR = 5% and RFF-only (i.e., no fall back to cryptographic authentication). The profile used for the energy measurement was 3 V, 2400 mAh battery, equivalent to two standard AA batteries. The MAC-only protocol showed an increased energy consumption of 24.4%, yielding a battery life decrease of almost 19.6% in comparison to the hybrid protocol configured for a simulated FRR = 10% as in Section 6, and which achieved a 99.8% total accuracy.

**Figure 11:**
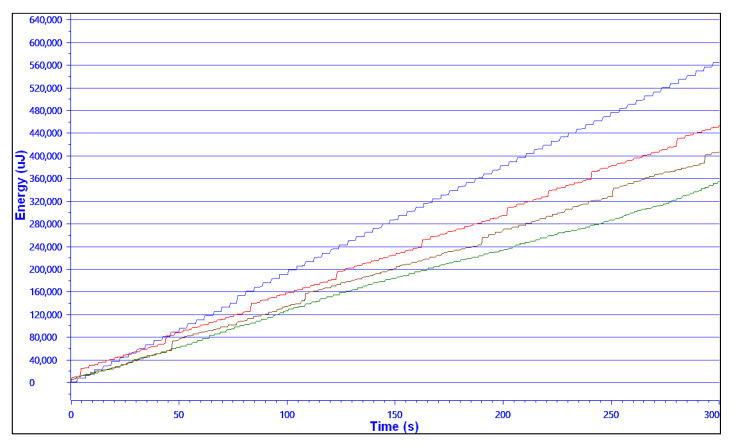
Energy measurement graph—From top to bottom: MAC-only (**blue**) vs. Hybrid FRR = 0.1 (**red**) vs. Hybrid FRR = 0.05 (**brown**) vs. RFF only (**green**).

Figure 11 shows the energy consumption graph of this setup over 5 min. Due to the first full authentication, the hybrid protocol was a little more energy-demanding at the start of the experiment. However, after only six transmissions, the total energy used by the MAC-only protocol was equivalent to that of the hybrid one, and after that, the advantage of the hybrid approach was apparent and grew almost linearly.

These results clearly show the value of the RFF as a primary method of authentication as part of a hybrid protocol, as defined in the first part of Section 4. In this energy consumption evaluation, we tried the hybrid system with values of the FRR configured to 10% and 5% to simulate recent RFF methods; any progress in the RFF technology allowing the use of lower FRR value without negatively affecting the FAR value will immediately drive significant energy savings for a given security level.

## 7. Conclusions

In this work, we described the need for lightweight and “good-enough” security for resource-starving devices. We proposed a protocol scheme able to leverage modern RF physical-layer-based fingerprinting methods and lightweight cryptographic solutions and to create a flexible hybrid message authentication scheme, without compromising the security level required. This scheme can save time and resources by using current RFF solutions, even if their intrinsic level of precision is not on par with the cryptographic-only methods. We evaluated this approach by precise energy level measurements, comparing the total energy consumption of a baseline cryptography-only authenticated protocol to a complete and fully real-time implementation of our hybrid scheme in IoT sensor nodes. The results provide a clear statement about the energy efficiency of this approach.

## Figures and Tables

**Table 1:** Energy measurement comparison.

System Name	MAC-Only	Hybrid w/FRR = 10%	Hybrid w/FRR = 5%	RFF-Only
**Time**	300 s	300 s	300 s	300 s
**Energy**	564.743 mJ	454.076 mJ	404.790 mJ	355.412 mJ
***Power***				
***Mean***	2.0922 mW	1.6342 mW	1.4625 mW	1.3449 mW
***Min***	0.0000 mW	0.0000 mW	0.0000 mW	0.0000 mW
***Max***	82.5882 mW	83.5259 mW	83.7475 mW	83.3862 mW
***Voltage***				
***Mean***	3.2798 V	3.2793 V	3.2793 V	3.2796 V
***Current***				
***Mean***	0.6373 mA	0.4980 mA	0.4457 mA	0.4111 mA
***Min***	0.0000 mA	0.0000 mA	0.0000 mA	0.0000 mA
***Max***	25.1640 mA	25.4552 mA	25.5250 mA	25.3826 mA
**Battery Life** (3 V, 2400 mAh)	5 months 7 days	6 months 15 days	7 months 9 days	8 months 2 days

## Abbreviations

The following abbreviations are used in this manuscript:

C&C	Command and control (center)
CRC	Cyclic redundancy check
DRBG	Deterministic random bit generator
EER	Equal error rate
FAR	False accept rate
FRR	False reject rate
GW	Gateway
ID	Identifier
IDS	Intrusion detection system
IoT	Internet of Things
KNN	k-nearest neighbors
LOS	Line-of-sight
MAC	Message authentication code
MITM	Man-in-the-middle
NLOS	Non-line-of-sight
PSK	Pre-shared key
RFF	Radio frequency fingerprinting
RSSI	Received signal strength indicator
SN	Sensor node
SNR	Signal-to-noise ratio
WSN	Wireless sensor network
